# Novel e4a2 BCR∷ABL1 transcript with insertion of CSE1L exons 9 and 10 in a CML patient: a case report

**DOI:** 10.3389/fonc.2025.1596256

**Published:** 2025-08-13

**Authors:** Sara Di Giusto, Eleonora Toffoletti, Cinzia Cozzarolo, Tomas Liani, Moira Moro, Renato Fanin, Daniela Damiani, Mario Tiribelli

**Affiliations:** ^1^ Division of Hematology and Stem Cell Transplantation, Udine Hospital, Udine, Italy; ^2^ Department of Medicine, Udine University, Udine, Italy

**Keywords:** chronic myeloid leukemia, atypical transcripts, tyrosine kinase inhibitors, outcome, *BCR::ABL1*

## Abstract

The *BCR::ABL1* fusion gene, resulting from the Philadelphia (Ph) chromosome, is the defining feature of Chronic Myeloid Leukemia (CML). The fusion transcript typically results from the juxtaposition of *ABL1* exons 2 or 3 and *BCR* exons 1, 13, 14 or 19, while exons 6 and 8 are less frequently involved. Here, we report the first case of a translocation in a patient with newly diagnosed chronic-phase CML harboring a novel e4a2 *BCR::ABL1* fusion gene. This unique fusion includes a 298 bp insertion, derived from a *CSE1L* gene exons 9 and 10, at the fusion site. The patient showed resistance to first-line dasatinib but achieved a molecular response with the third-generation tyrosine kinase inhibitor ponatinib.

## Introduction

The pathognomonic marker of chronic myeloid leukemia (CML) is the well-known cytogenetic aberration defined as the Philadelphia (Ph) chromosome (1). The Ph chromosome results from a reciprocal translocation involving the 3’ region of the *ABL1* proto-oncogene (9q34) and the 5’region of the *BCR* gene (22q11). The *BCR::ABL1* oncogene encodes a constitutively active tyrosine kinase that promotes leukemogenesis (2).

The reciprocal translocation results in a breakpoint within the *ABL1* gene, specifically in the region located between exons 1a, 1b and 2. Breakpoints on the *BCR* gene can occur in various regions, leading to the formation of different fusion genes that encode distinct transcripts and three main proteins: e13a2 and e14a2 (both producing the p210 protein), e1a2 (producing the p190 protein) and e19a2 (producing the p230 protein). These proteins share structural similarities, but exhibit different properties. Notably, the amount of *BCR* sequence included in the fusion gene affects the tyrosine kinase activity of the protein: p190 demonstrates higher activity than p210, which in turn has higher activity than p230 (3).

Recently, atypical *BCR* breakpoints have been reported, leading to the formation of different fusion genes such as e1a3, e13a3, e14a3 e19a3, e6a2, e8a2 and e15a2. These involve splicing between whole exons, insertion of small sequences, or genomic breakpoints within exons (4). The most frequent breakpoint regions in the *ABL1* gene are located 5’ of the second exon, resulting in a2 junctions. Other breakpoint regions have been observed between the second and the third exons, leading to a3 junctions (5). To date, no atypical breakpoints have been identified within the *ABL1* (6).

CSE1L (Chromosome Segregation 1-Like), also known as CAS (Cellular Apoptosis Susceptibility), is a pleiotropic protein that plays a role in apoptosis, cell survival, chromosome assembly, nucleocytoplasmic transport, microvesicle formation and cancer metastatic dissemination. The phosphorylation status of CSE1L is modulated by the Extracellular signal-Regulated Kinase (ERK). Conversely, CSE1L can influence RAS-induced ERK phosphorylation and the expression and phosphorylation of key transcription factors CREB (cAMP Response Element Binding) and MITF (Microphtalmia-associated Transcription Factor) (7).

The *CSE1L* gene is located on chromosome 20q13.13 and its expression has been detected in the bone marrow of CML patients and in K562 cells. Its knockdown resulted in cell cycle arrest at the G0/G1 phase, preventing entry into the S phase, and leading to apoptosis. Furthermore, CSE1L increases phosphorylated AMPK and decreases phosphorylated mTOR, playing a critical role in K562 cell survival and growth. Instead, CSE1L downregulation is associated with the cytotoxic effects of imatinib in CML patients (8).

## Case description

A 41-year-old man was referred to the Department of Hematology at Udine University Hospital in April 2017, following the detection of thrombocytosis during a preoperative routine assessment. Peripheral blood cell analysis confirmed thrombocytosis (platelet count: 910 x 10^9^/L), and revealed mild leukocytosis (white blood cell count: 14.3 x 10^9^/L), with a differential count of 79% neutrophils, 18% lymphocytes, and 3% eosinophils. No circulating myeloid progenitors were observed. Physical examination revealed no superficial lymphadenopathy or organomegalies, though the patient exhibited facial erythema and round, erythematous lesions on the neck.

**Figure 1:**
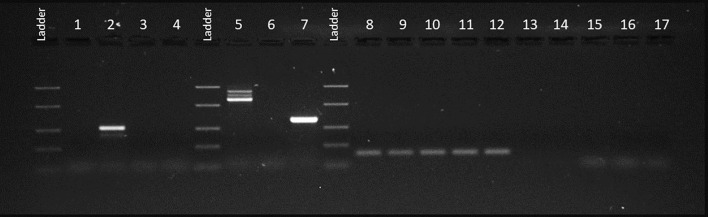
UV gel image of PCR products using BIOMED-1 primers. PCR products using *BCR::ABL1* e13a3 primers: our case (1), p210 Positive Control (PC) (2), p210 Negative Control (NC) (3), PCR-No Template Control (NTC) (4) and RT-NTC (17). PCR products using *BCR∷ABL1* e1a3 primes: our case (5), p190 PC (7), p190 NC (6), RT-NTC (15) and PCR-NTC (16). PCR products using *ABL1* primers: our case (8), p210 PC (9), p210 NC (10), p190 PC (11), p190 NC (12), RT-NTC (13) and PCR-NTC (14).

Due to the suspicion of a myeloproliferative neoplasm, the patient underwent a bone marrow biopsy and conventional cytogenetic analysis. The cytogenetic evaluation revealed a variant Philadelphia chromosome in 100% of analyzed metaphases. Fluorescent *in situ* hybridization (FISH) using a *BCR::ABL1*-specific probe showed an interstitial deletion in 9q34. *JAK2* V617F mutation analysis and reverse transcription polymerase chain reaction (RT-PCR) for *BCR::ABL1* fusion transcripts were also performed. *JAK2* V617F mutation analysis yielded negative results. Conversely, BIOMED-1 RT-PCR, optimized for the detection of the e13a2, e14a2 and e1a2 isoforms of the *BCR::ABL1* fusion gene (9), revealed two atypical amplification products migrating between 1300 bp and 1000 bp upon analysis by 2% agarose gel electrophoresis and visualization under UV illumination, specifically when using e1a3 primers (**Figure 1**).

The first PCR product was sequenced (BigDye™ Terminator v3.1 Cycle Sequencing Kit – Applied Biosystems) with the same primers as the RT-PCR. The forward sequence obtained from the PCR product revealed a coherent open reading frame extending to *BCR* exon 4, followed by a segment that overlaps with an additional sequence. A comparable, overlapping pattern was observed in the reverse sequence, starting from the beginning of *ABL1* exon 2.

**Figure 2:**
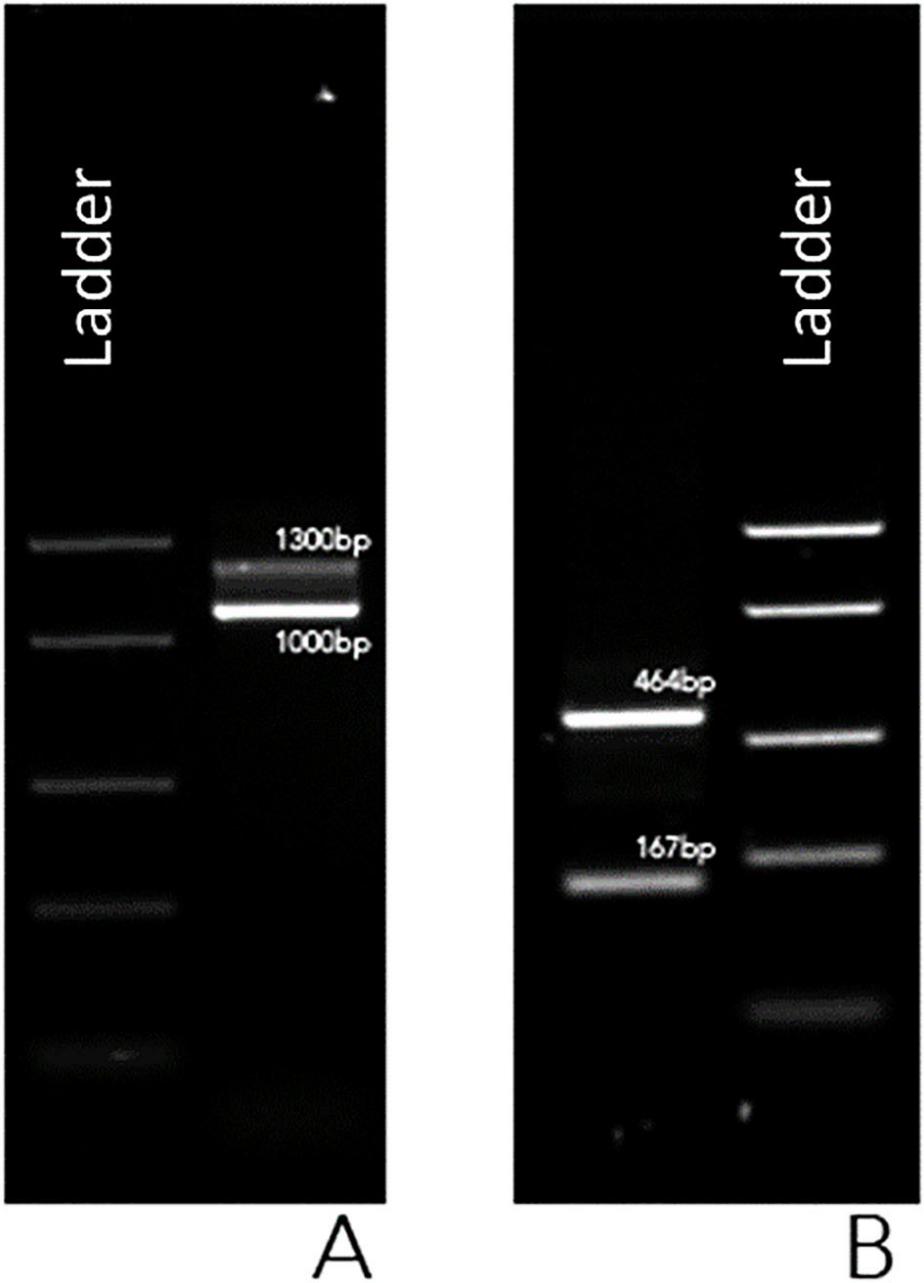
UV gel image of PCR products using BIOMED-1 *BCR::ABL1* e1a3 primers **(A)** and using custom-designed primers in *BCR* exon 4 and in *ABL1* exon 2 **(B)**.

To further characterize the fusion transcript, novel oligonucleotide primers were designed to anneal to *BCR* exon 4 (5’-GCTCTACGAGATCCACAAGGAG-3’) and *ABL1* exon 2 (5’-GAGTTCCAACGAGCGGCTT-3’). The *BCR::ABL1* fusion transcript was subsequently screened following the established protocol. The resulting PCR amplicons were analyzed by 1% agarose gel electrophoresis and visualized under UV illumination, revealing two distinct amplification products, one measuring 464 bp and the other measuring 167 bp (**Figure 2**).

**Figure 3:**
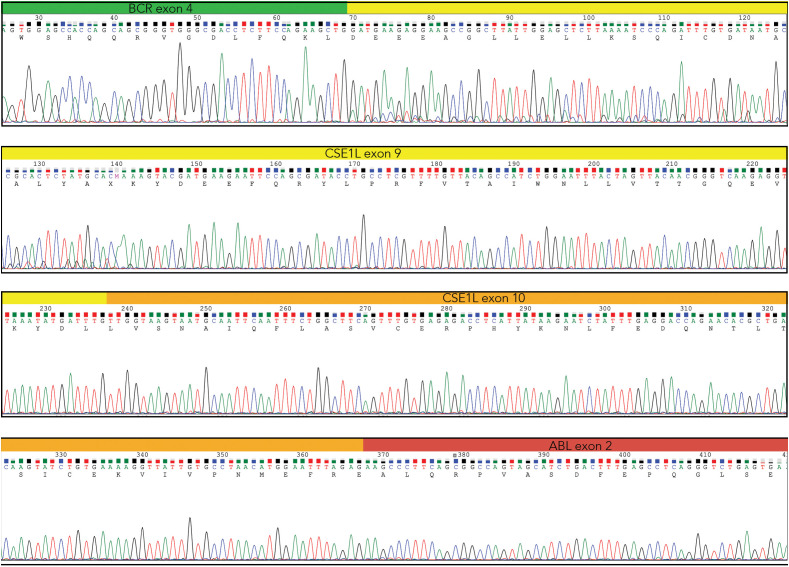
Direct sequencing of the Long Fragment showing *BCR* exon 4 (green) and *ABL1* exon2 (red), with the insertion of the *CSE1L* exons 9 (yellow) and 10 (orange) (Chromas – Technelysium DNA Sequencing Software – ).

Following gel extraction and purification, both DNA fragments underwent Sanger sequencing. Bidirectional sequencing of the Short Fragment (SF) identified the fusion junction between *BCR* exon 4 and the *ABL1* exon 2. Likewise, the forward and reverse sequences of the Long Fragment (LF) displayed the same breakpoint (e4a2) as the SF, along with an insertion of *CSE1L* (Chromosome Segregation 1 Like) gene exons 9 and 10 at the fusion site (**Figure 3**).

The incorporation of *CSE1L* exons within the LF allows the fusion transcript to maintain an open reading frame throughout its entire length, leading us to assume that this transcript encodes the disease-relevant protein. Conversely, the SF harbors a premature stop codon at nucleotide position 1773 (located 18 bp downstream of the 5’ end of *ABL1* exon 2), predicted to yield a truncated protein that is likely degraded.

Furthermore, the LF, incorporating the *CSE1L* exons, maintains critical functional domains, including the ATP Binding Site – the pharmacological target of prevalent Tyrosine Kinase Inhibitors (TKIs) such as imatinib, nilotinib and ponatinib – and the Myristoyl Binding Site, the target of the recently developed allosteric TKI asciminib (10).

The final diagnosis was of chronic-phase CML with atypical *BCR::ABL1* transcript, classified as low risk according to both Sokal and ELTS scoring systems. Due to the patient’s young age and the presence of this atypical *BCR::ABL1* transcript, he was considered eligible for front-line therapy with a second-generation TKI and began taking dasatinib at a dose of 100 mg/day in July 2017.

The patient achieved a complete hematologic response by the first month of treatment; however, grade 2–3 thrombocytopenia (platelet count: approximately 50–60 x 10^9^/L) developed, prompting a temporary reduction of the dasatinib daily dose to 50 mg. Cytogenetic analysis performed after 3 months of dasatinib therapy revealed 45% Ph+ metaphases, decreasing to 10% at 6 months. Cytogenetic testing 12 months after starting dasatinib showed the persistence of Ph+ in 10% of metaphases and the emergence of two new Ph-negative clones: one with trisomy of chromosome 8 (15% of metaphases) and one with monosomy of chromosome 7 (75% of metaphases). The patient maintained a good clinical status despite persistent thrombocytopenia (platelet count: 55 x 10^9^/L) and neutropenia (neutrophil count: 0.8 x 10^9^/L).

Failure to achieve a complete cytogenetic response (CCyR) by 12 months, coupled with the appearance of Additional Cytogenetic Abnormalities (ACA) within the Ph- clone, including -7 in 75% of metaphases, resulted in the discontinuation of dasatinib therapy. In October 2018, treatment was switched to a second-line treatment with ponatinib 45 mg/day. The patient’s only sibling refused HLA typing.

Ponatinib treatment was well tolerated, although the dose was reduced to 30 mg/day after 2 months due to persistent thrombocytopenia (platelet count: 60–90 x 10^9^/L) and mild neutropenia (neutrophil count: 1.0-1.2 x 10^9^/L). After 6 months of ponatinib therapy, the patient achieved a CCyR with a reduction of the -7-clone to 10%, while the +8-clone increased to 65%. The CCyR was sustained at the 12-month of ponatinib therapy, with complete disappearance of the -7 clone. These findings were further validated at 3- and 4-years follow-ups after ponatinib therapy (regularly taken at 30 mg/day), with 0% Ph+, no monosomy 7, and trisomy 8 present in 80% and 60% of metaphases, respectively. After 1 year of therapy, the cytopenias began to improve. By 4th year after ponatinib switch, the patient reached a normal blood count with hemoglobin: 15.6 g/dL, platelet count: 159 x 10^9^/L, white blood cell count: 5,7 x 10^9^/L with a preserved formula.

## Discussion

The relationship between the e4a2 variant transcript and treatment prognosis has not yet been established. It is also debated how this variant modulates the CML phenotype. In this case, cytogenetic analysis was performed due to the inability to quantify *BCR::ABL1* transcript levels using qPCR.

The patient did not achieve a CCyR with first-line dasatinib therapy, whereas this goal was reached after 6 months of ponatinib therapy, which was maintained thereafter. Similarly, under ponatinib therapy, we observed clearance of one of the ACAs, specifically monosomy of chromosome 7.

Molecular monitoring was performed using a semi-quantitative assay based on the BIOMED-1 methodology, analyzing both first- and nested-PCR amplicons. The results were compared to a standard curve generated through stepwise dilutions of the diagnostic specimen. Molecular analysis revealed the presence of the two transcript sequences at 6, 12 and 18 months after initiating ponatinib therapy. The transcripts disappeared at 24 months of ponatinib therapy, in both the first- and the nested-PCR analyses (9). At the last follow-up, 7 years after diagnosis, the LF reappeared in nested-PCR only. These evaluations of minimal residual disease were performed in accordance with the LeukemiaNet 2020 (11) and GIMEMA recommendations.

The peculiar nature of the e4a2 transcript poses a challenge for assessing long-term prognosis and therapeutic response. As evidenced by this case, the patient did not achieve an optimal response with front-line dasatinib, requiring a switch to ponatinib. Furthermore, the lack of sufficiently sensitive data for minimal residual disease precludes the evaluation of a stable deep molecular response, a prerequisite for potential TKI therapy discontinuation.

Schäfer et al. suggested monitoring the molecular responses of CML patients with atypical *BCR::ABL1* fusion transcripts by RT-qPCR with specific primers and probes, using serial dilutions of *BCR::ABL1* and *GUSβ* calibrators. In our case, the e4a2 transcript represents a novel variant for which no established calibrator is available (12).

One potential solution to the lack of a standardized test for CML monitoring could be the development of a patient-specific assay using optimized digital-PCR probes. This approach could enable the transition from a semi-quantitative assessment to absolute quantification, thus eliminating the need for an initial dilution curve. Unfortunately, in this particular case, all available pathological material has been exhausted, precluding the completion of this assay.

The presence of only two exons of the *CSE1L* gene likely does not affect the tyrosine kinase properties of the *BCR::ABL1* fusion protein. However, it is interesting that the silencing of CSE1L protein has been implicated with modulating proliferation and apoptosis through the AMPK/mTOR signaling pathway in CML cell lines (8). This observation suggests a more significant interaction between these two proteins in the pathogenesis of the disease.

## Data Availability

The datasets presented in this study can be found in online repositories. The names of the repository/repositories and accession number(s) can be found below: https://www.ncbi.nlm.nih.gov/genbank/, PV263178.
